# Supplementary material for: Autophagy Triggers Tamoxifen Resistance in Human Breast Cancer Cells by Preventing Drug-Induced Lysosomal Damage
Source: Cancers (Basel). 2021 Mar 12;13(6):1252. doi: 10.3390/cancers13061252 (PMC7999102; doi:10.3390/cancers13061252)
Supplement: Supplementary file 1 [file cancers-13-01252-s001.pptx]

## Slide 1
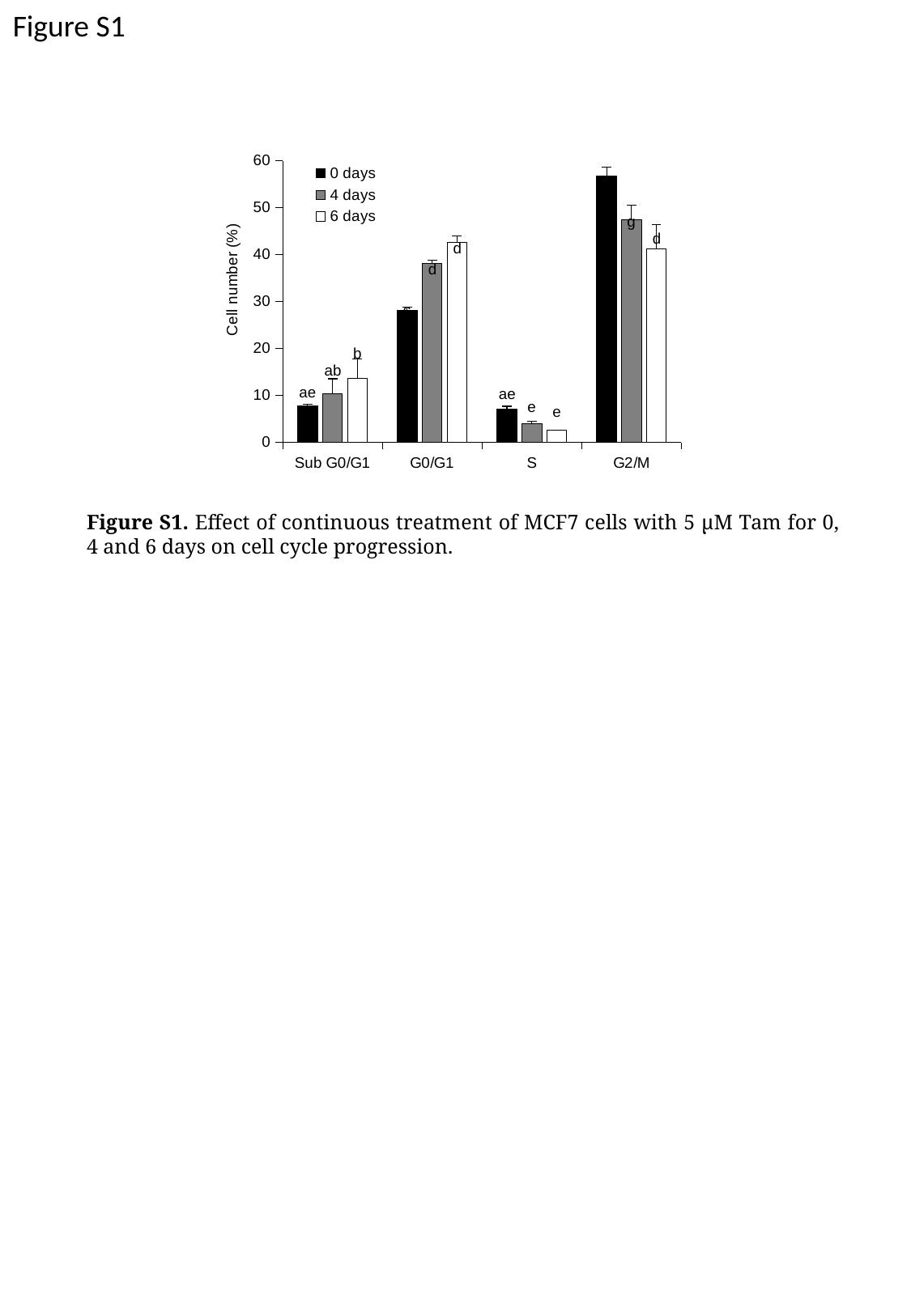

Figure S1
### Chart
| Category | | | |
|---|---|---|---|
| Sub G0/G1 | 7.8 | 10.350000000000003 | 13.65 |
| G0/G1 | 28.1 | 38.1 | 42.650000000000006 |
| S | 7.05 | 4.050000000000001 | 2.55 |
| G2/M | 56.7 | 47.5 | 41.25 |f
g
d
d
d
c
b
ab
ae
ae
e
e
Figure S1. Effect of continuous treatment of MCF7 cells with 5 µM Tam for 0, 4 and 6 days on cell cycle progression.

## Slide 2
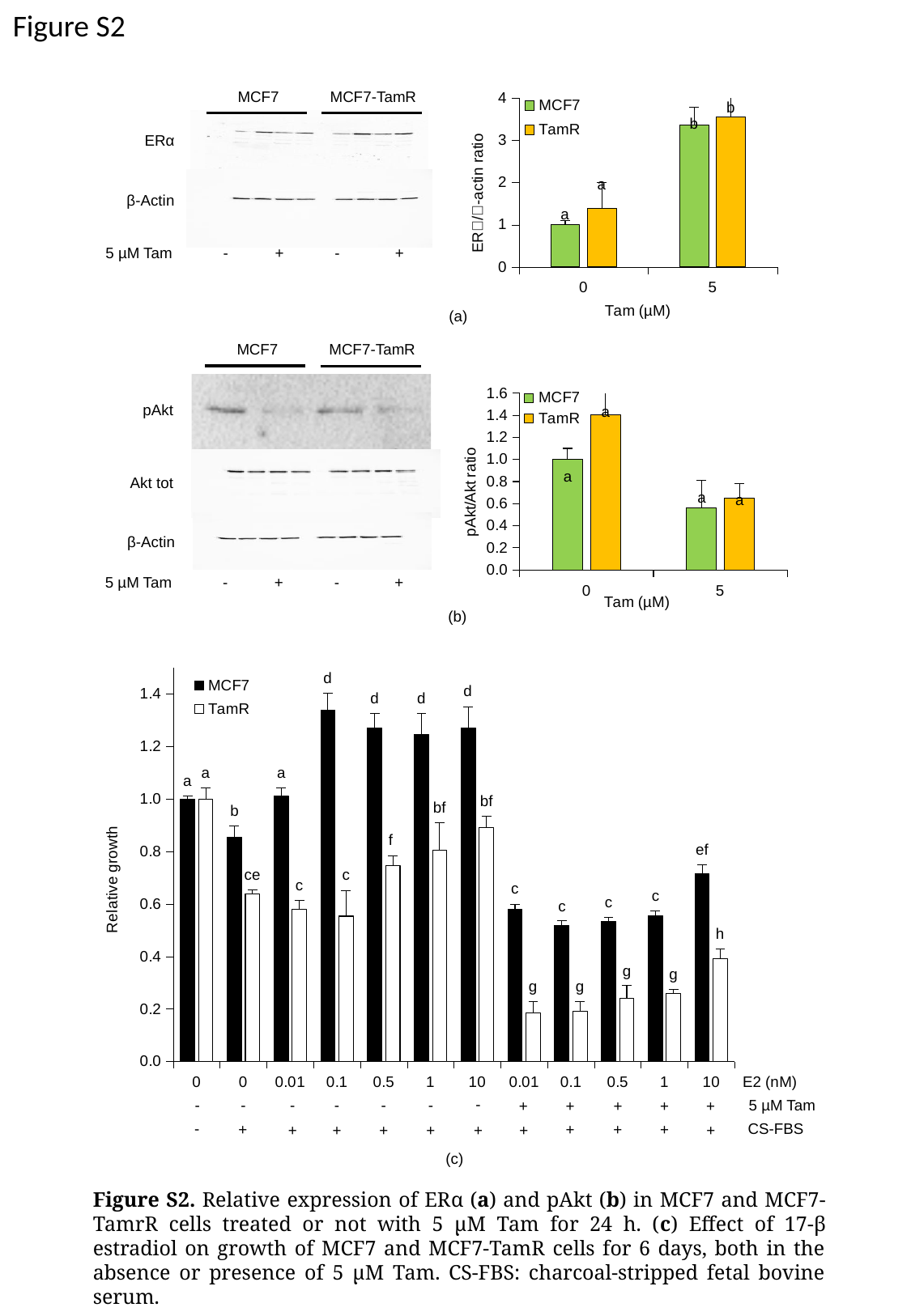

Figure S2
### Chart
| Category | | |
|---|---|---|
| 0 | 1.0 | 1.3852535066048721 |
| 5 | 3.362626357915128 | 3.5609118777529294 | MCF7 MCF7-TamR
ERα
β-Actin
5 µM Tam - + - +
b
b
a
a
(a)
 MCF7 MCF7-TamR
pAkt
Akt tot
β-Actin
5 µM Tam - + - +
### Chart
| Category | | |
|---|---|---|
| 0 | 1.0 | 1.4073484355096764 |
| 5 | 0.5610826046872278 | 0.6473675355579008 |a
a
a
a
(b)
### Chart
| Category | | |
|---|---|---|
| 0 | 1.0 | 1.0 |
| 0 | 0.8538975501113585 | 0.6385393535652604 |
| 0.01 | 1.0120267260579063 | 0.5812978040957315 |
| 0.1 | 1.3389755011135858 | 0.5536639526276832 |
| 0.5 | 1.2694877505567927 | 0.7453737971872687 |
| 1 | 1.246325167037862 | 0.8065630397236615 |
| 10 | 1.2703786191536748 | 0.8902047865778435 |
| 0.01 | 0.579510022271715 | 0.18430792005921537 |
| 0.1 | 0.5180400890868597 | 0.19170984455958548 |
| 0.5 | 0.533630289532294 | 0.24031581544534913 |
| 1 | 0.5541202672605791 | 0.2605477424130274 |
| 10 | 0.7149220489977728 | 0.3913150752528991 |d
d
d
d
a
a
a
bf
bf
b
f
ef
ce
c
c
c
c
c
c
h
g
g
g
g
-
-
-
-
-
-
-
5 µM Tam
+
+
+
+
+
-
CS-FBS
+
+
+
+
+
+
+
+
+
+
+
(c)
Figure S2. Relative expression of ERα (a) and pAkt (b) in MCF7 and MCF7-TamrR cells treated or not with 5 µM Tam for 24 h. (c) Effect of 17-β estradiol on growth of MCF7 and MCF7-TamR cells for 6 days, both in the absence or presence of 5 µM Tam. CS-FBS: charcoal-stripped fetal bovine serum.

## Slide 3
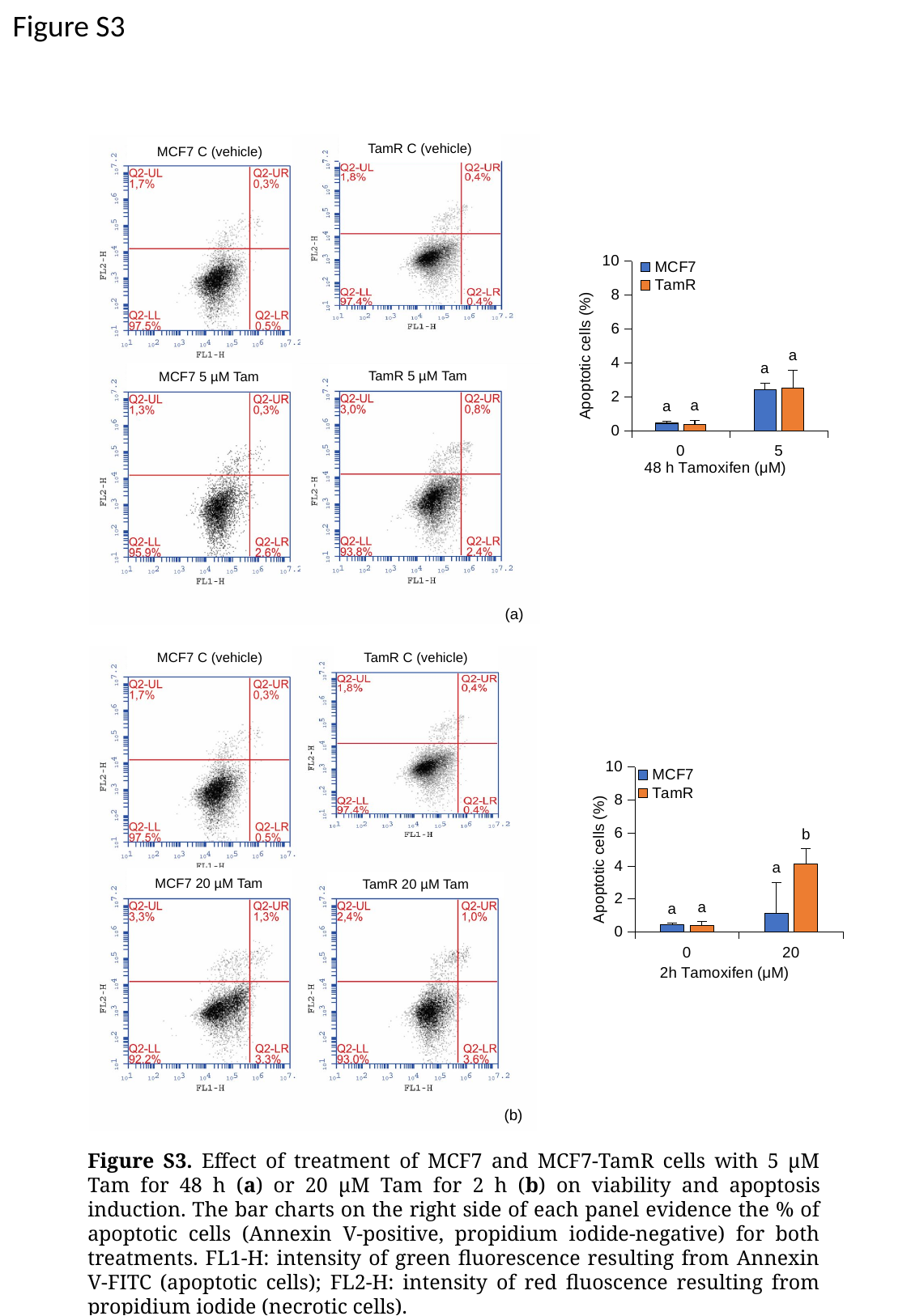

Figure S3
TamR C (vehicle)
MCF7 C (vehicle)
### Chart
| Category | | |
|---|---|---|
| 0 | 0.43333333333333335 | 0.3666666666666667 |
| 5 | 2.433333333333333 | 2.5 |a
a
TamR 5 µM Tam
MCF7 5 µM Tam
a
a
(a)
MCF7 C (vehicle)
TamR C (vehicle)
### Chart
| Category | | |
|---|---|---|
| 0 | 0.43333333333333335 | 0.3666666666666667 |
| 20 | 1.1333333333333333 | 4.133333333333334 |b
a
MCF7 20 µM Tam
TamR 20 µM Tam
a
a
(b)
Figure S3. Effect of treatment of MCF7 and MCF7-TamR cells with 5 µM Tam for 48 h (a) or 20 µM Tam for 2 h (b) on viability and apoptosis induction. The bar charts on the right side of each panel evidence the % of apoptotic cells (Annexin V-positive, propidium iodide-negative) for both treatments. FL1-H: intensity of green fluorescence resulting from Annexin V-FITC (apoptotic cells); FL2-H: intensity of red fluoscence resulting from propidium iodide (necrotic cells).

## Slide 4
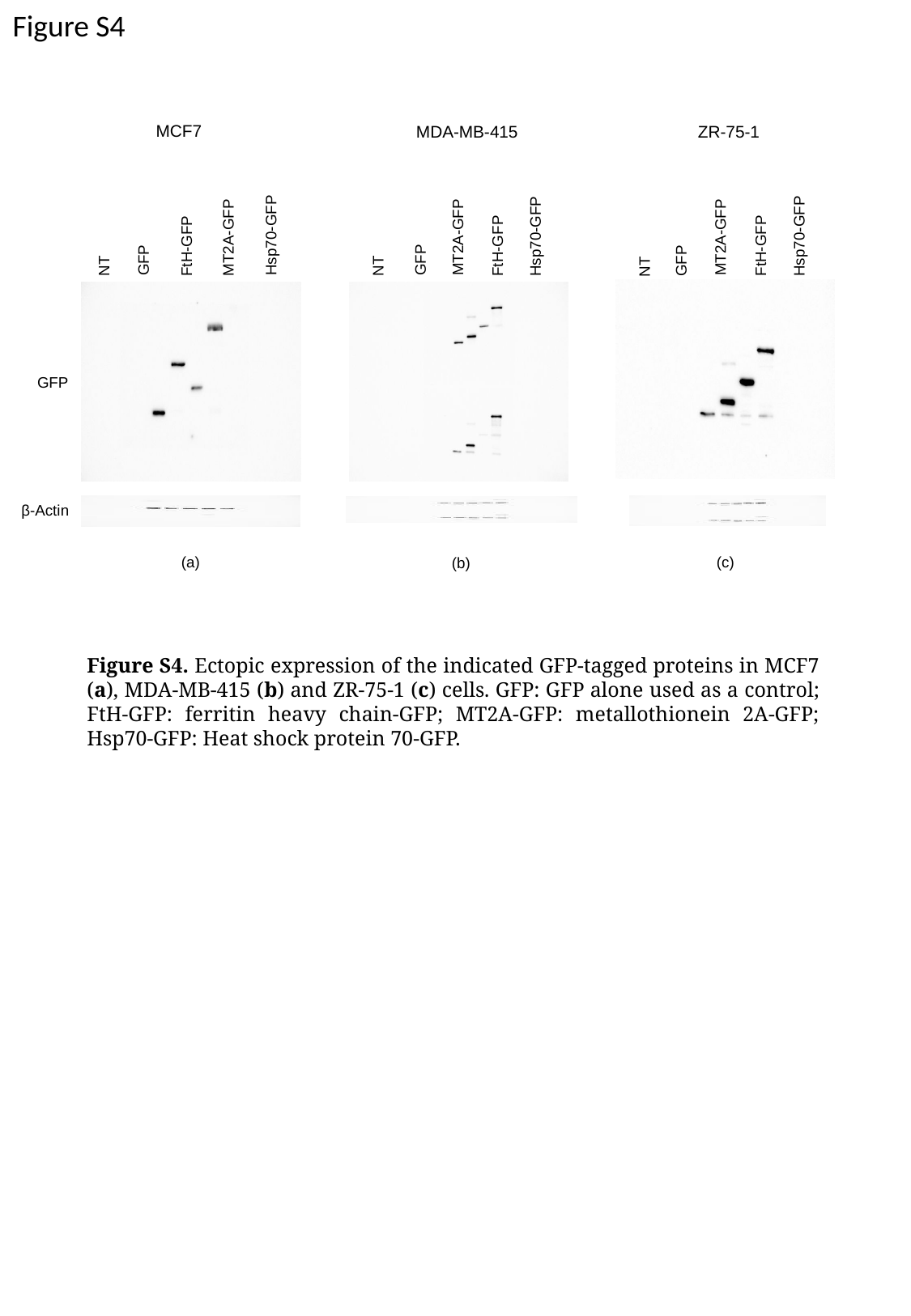

Figure S4
MCF7
Hsp70-GFP
MT2A-GFP
FtH-GFP
GFP
NT
(a)
MDA-MB-415
Hsp70-GFP
MT2A-GFP
FtH-GFP
GFP
NT
(b)
ZR-75-1
Hsp70-GFP
MT2A-GFP
FtH-GFP
GFP
NT
(c)
GFP
β-Actin
Figure S4. Ectopic expression of the indicated GFP-tagged proteins in MCF7 (a), MDA-MB-415 (b) and ZR-75-1 (c) cells. GFP: GFP alone used as a control; FtH-GFP: ferritin heavy chain-GFP; MT2A-GFP: metallothionein 2A-GFP; Hsp70-GFP: Heat shock protein 70-GFP.

## Slide 5
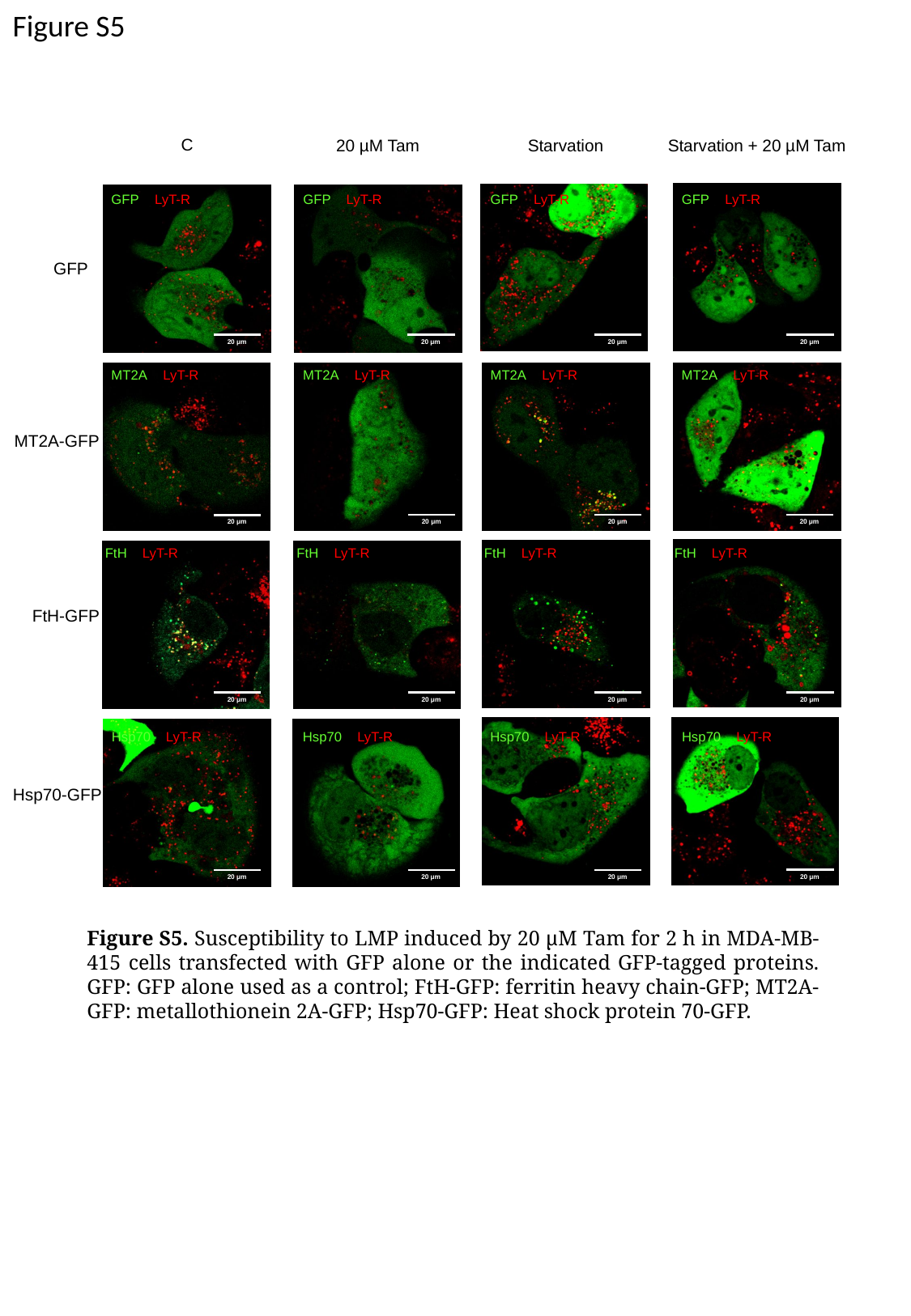

Figure S5
C
Starvation + 20 µM Tam
20 µM Tam
Starvation
GFP LyT-R
GFP LyT-R
GFP LyT-R
GFP LyT-R
GFP
20 μm
20 μm
20 μm
20 μm
MT2A LyT-R
MT2A LyT-R
MT2A LyT-R
MT2A LyT-R
MT2A-GFP
20 μm
20 μm
20 μm
20 μm
FtH LyT-R
FtH LyT-R
FtH LyT-R
FtH LyT-R
FtH-GFP
20 μm
20 μm
20 μm
20 μm
Hsp70 LyT-R
Hsp70 LyT-R
Hsp70 LyT-R
Hsp70 LyT-R
Hsp70-GFP
20 μm
20 μm
20 μm
20 μm
Figure S5. Susceptibility to LMP induced by 20 µM Tam for 2 h in MDA-MB-415 cells transfected with GFP alone or the indicated GFP-tagged proteins. GFP: GFP alone used as a control; FtH-GFP: ferritin heavy chain-GFP; MT2A-GFP: metallothionein 2A-GFP; Hsp70-GFP: Heat shock protein 70-GFP.

## Slide 6
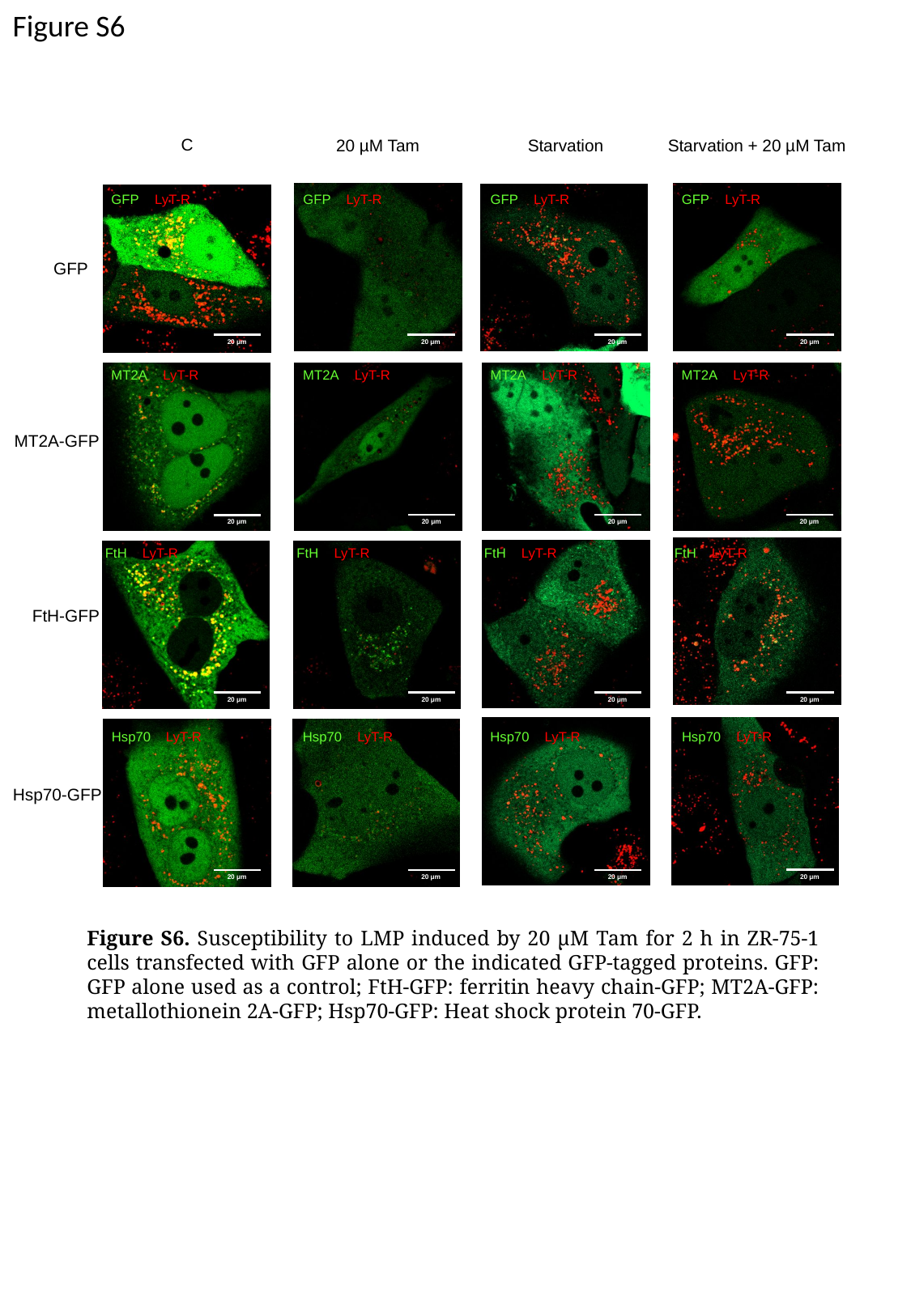

Figure S6
C
Starvation + 20 µM Tam
20 µM Tam
Starvation
GFP LyT-R
GFP LyT-R
GFP LyT-R
GFP LyT-R
GFP
20 μm
20 μm
20 μm
20 μm
MT2A LyT-R
MT2A LyT-R
MT2A LyT-R
MT2A LyT-R
20 μm
20 μm
20 μm
20 μm
FtH LyT-R
FtH LyT-R
FtH LyT-R
FtH LyT-R
20 μm
20 μm
20 μm
20 μm
Hsp70 LyT-R
Hsp70 LyT-R
Hsp70 LyT-R
Hsp70 LyT-R
20 μm
20 μm
20 μm
20 μm
MT2A-GFP
FtH-GFP
Hsp70-GFP
Figure S6. Susceptibility to LMP induced by 20 µM Tam for 2 h in ZR-75-1 cells transfected with GFP alone or the indicated GFP-tagged proteins. GFP: GFP alone used as a control; FtH-GFP: ferritin heavy chain-GFP; MT2A-GFP: metallothionein 2A-GFP; Hsp70-GFP: Heat shock protein 70-GFP.

## Slide 7
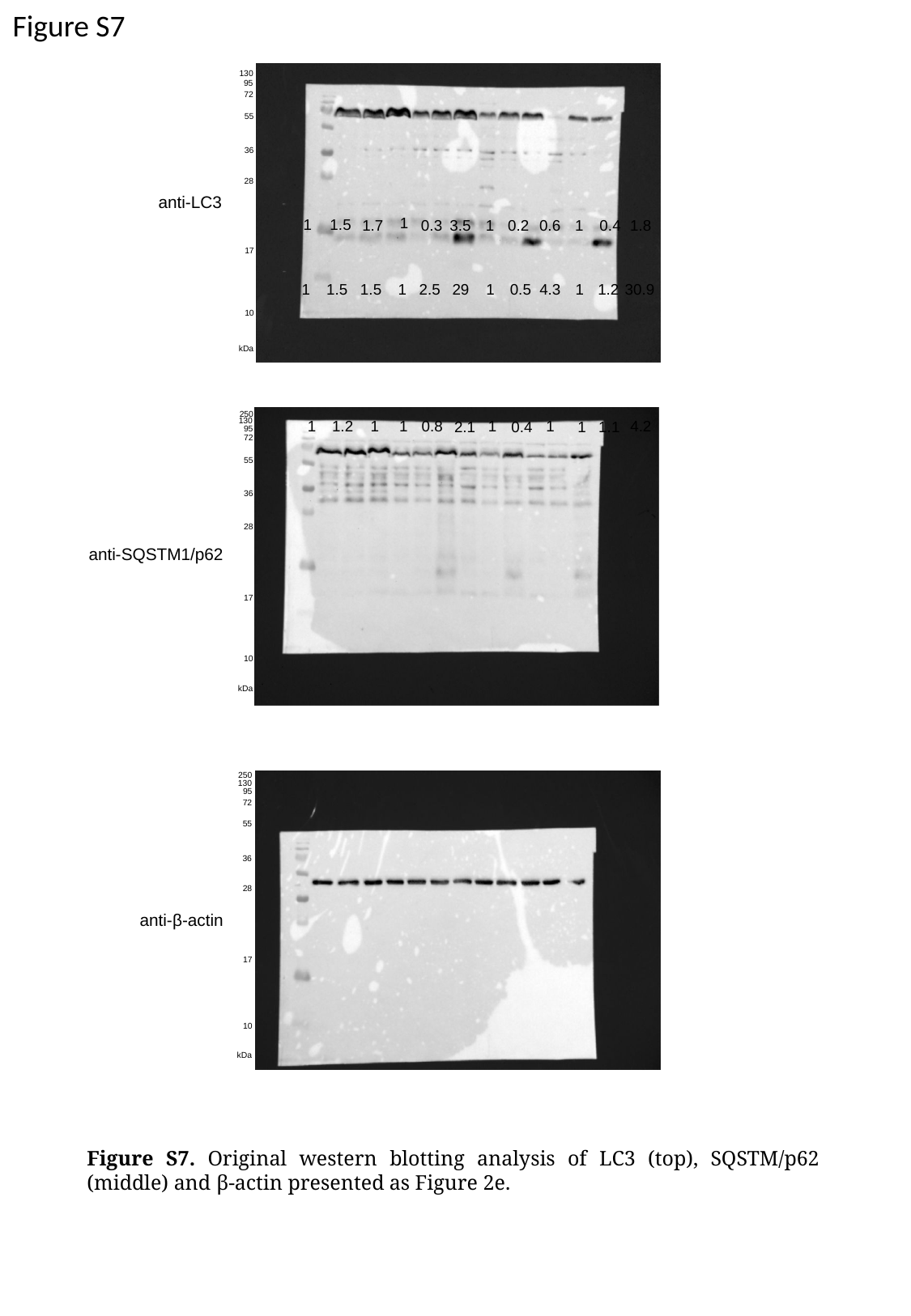

Figure S7
130
95
72
55
36
28
17
10
kDa
anti-LC3
1
1
1.5
1.7
0.3
3.5
1
0.2
0.6
1
0.4
1.8
1
1.5
1.5
1
2.5
29
1
0.5
4.3
1
1.2
30.9
250
130
95
72
55
36
28
17
10
kDa
4.2
1
1
1
1
1.2
1
0.8
2.1
1
1.1
0.4
anti-SQSTM1/p62
250
130
95
72
55
36
28
17
10
kDa
anti-β-actin
Figure S7. Original western blotting analysis of LC3 (top), SQSTM/p62 (middle) and β-actin presented as Figure 2e.

## Slide 8
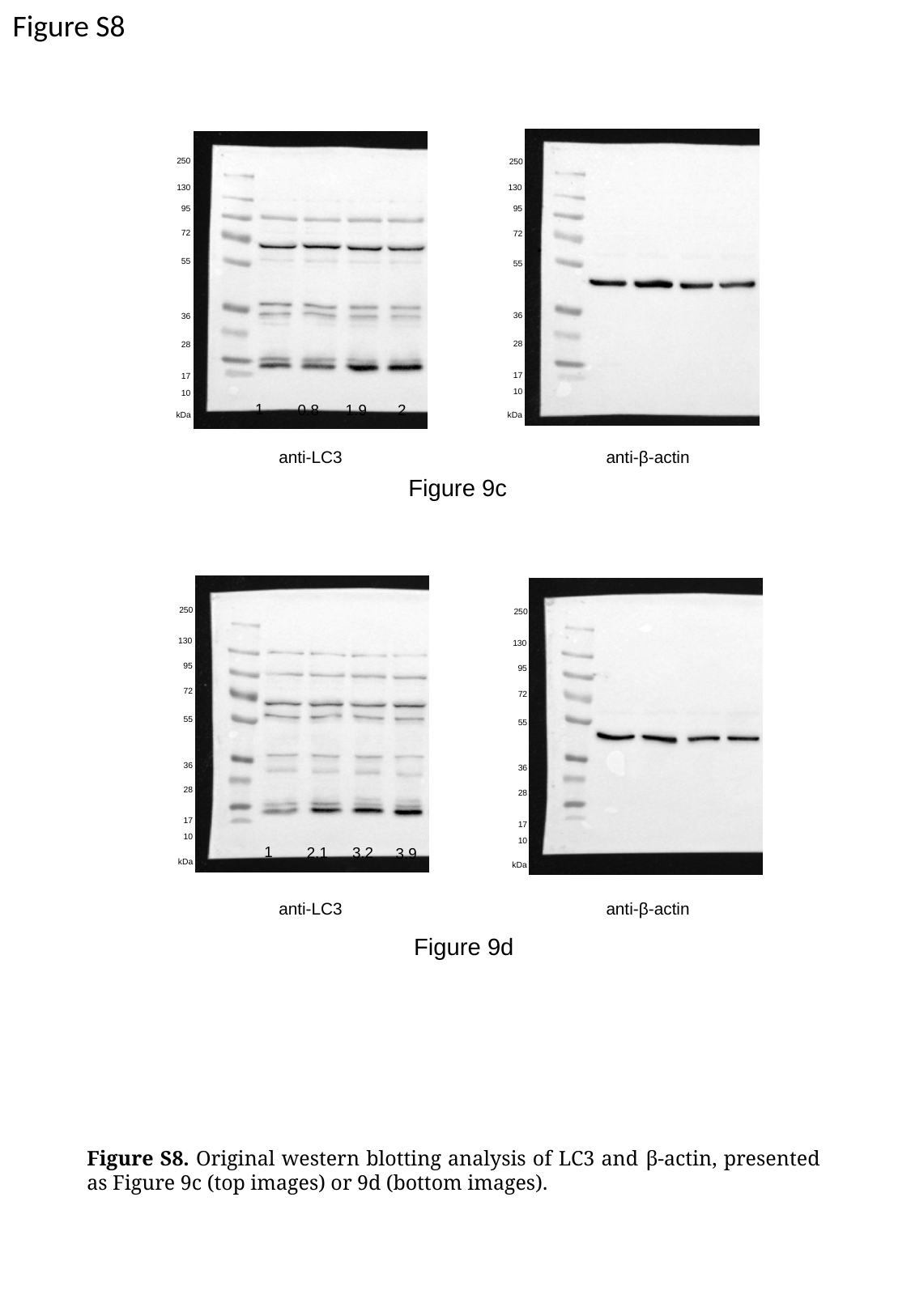

Figure S8
250
130
95
72
55
36
28
17
10
kDa
anti-β-actin
250
130
95
72
55
36
28
17
10
kDa
1
0.8
1.9
2
anti-LC3
Figure 9c
250
130
95
72
55
36
28
17
10
kDa
1
2.1
3.2
3.9
anti-LC3
250
130
95
72
55
36
28
17
10
kDa
anti-β-actin
Figure 9d
Figure S8. Original western blotting analysis of LC3 and β-actin, presented as Figure 9c (top images) or 9d (bottom images).

## Slide 9
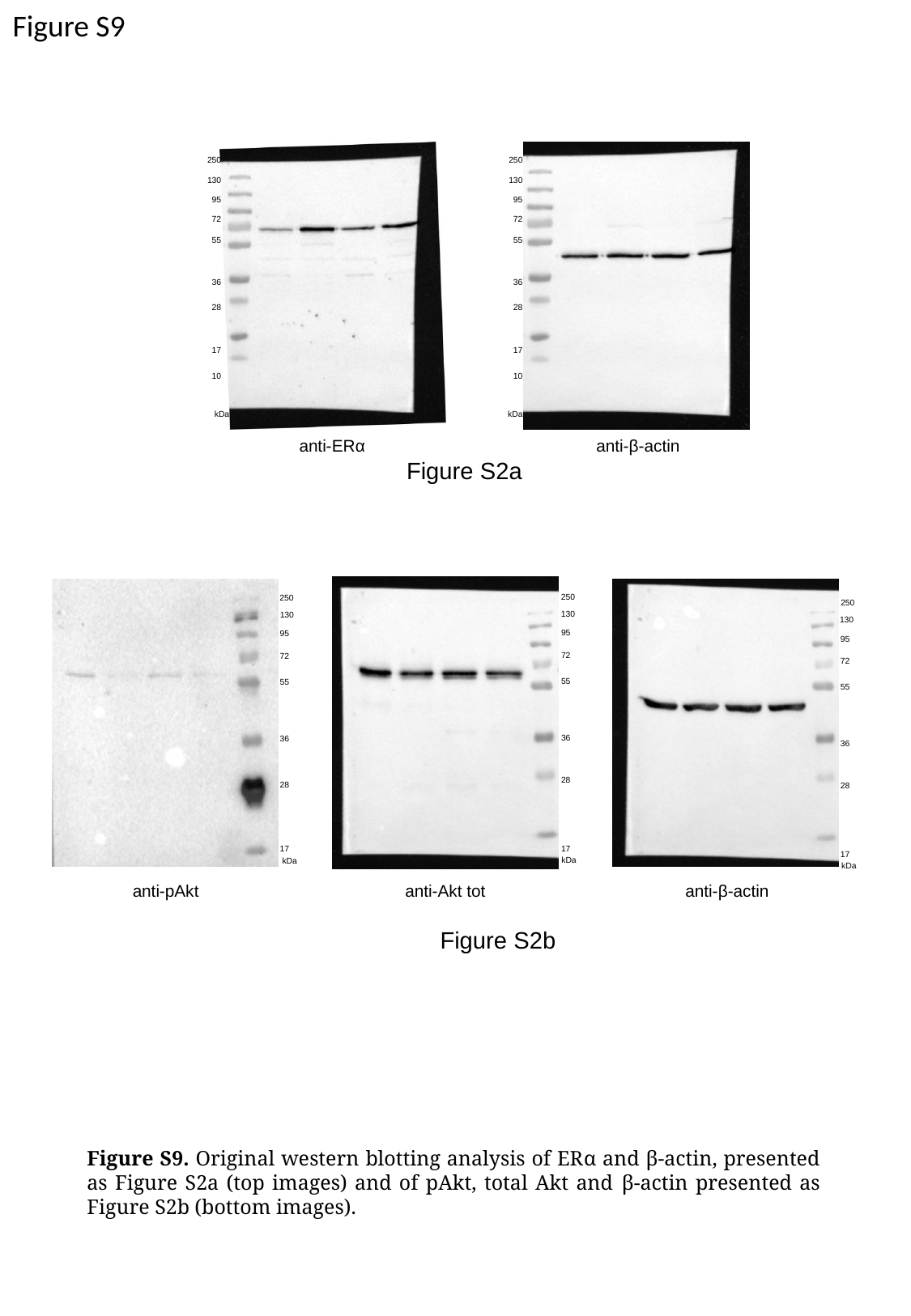

Figure S9
250
130
95
72
55
36
28
17
10
kDa
250
130
95
72
55
36
28
17
10
kDa
anti-β-actin
anti-ERα
Figure S2a
250
130
95
72
55
36
28
17
kDa
250
130
95
72
55
36
28
17
kDa
250
130
95
72
55
36
28
17
kDa
anti-β-actin
anti-Akt tot
anti-pAkt
Figure S2b
Figure S9. Original western blotting analysis of ERα and β-actin, presented as Figure S2a (top images) and of pAkt, total Akt and β-actin presented as Figure S2b (bottom images).

## Slide 10
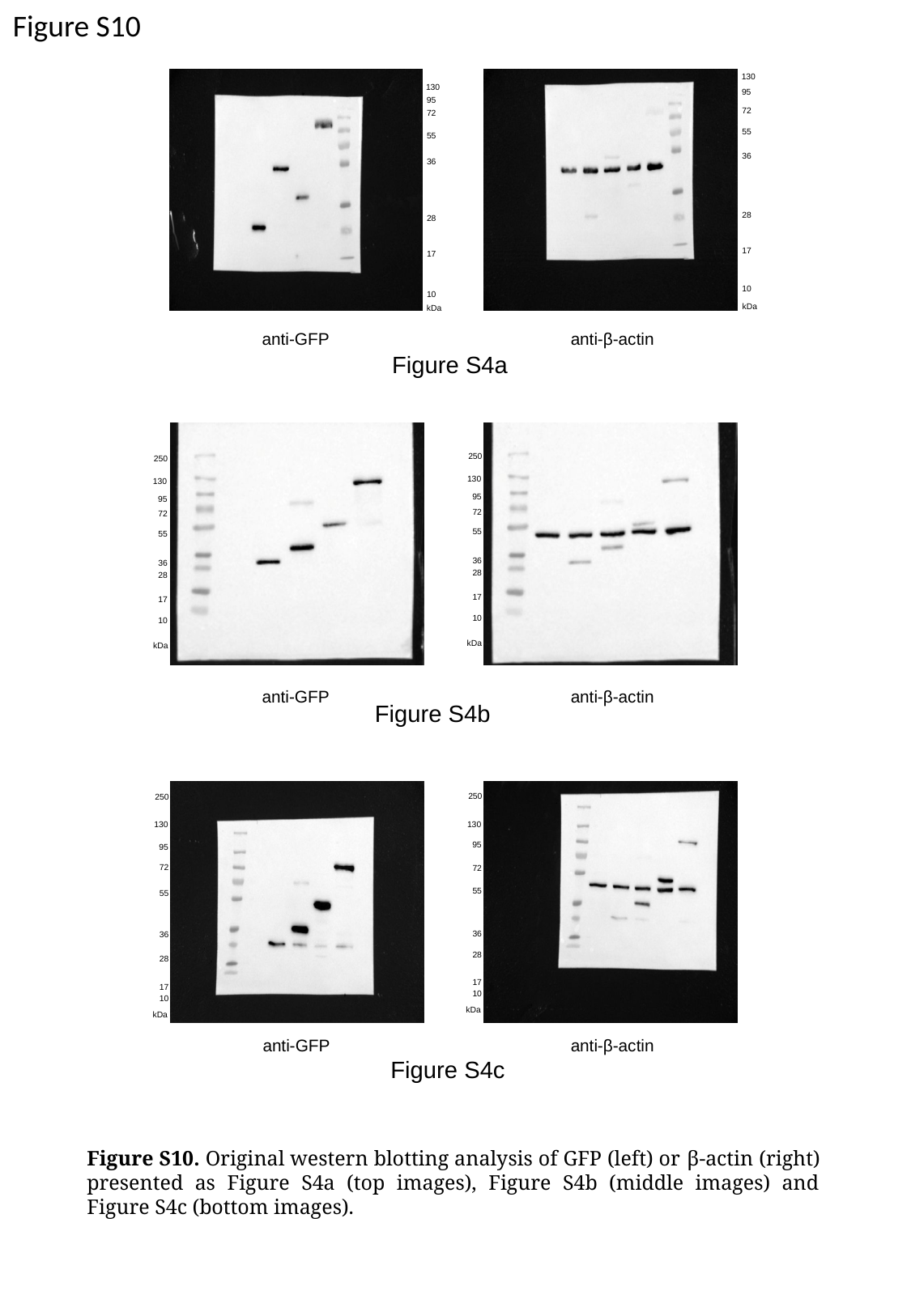

Figure S10
130
95
72
55
36
28
17
10
kDa
130
95
72
55
36
28
17
10
kDa
anti-GFP
anti-β-actin
Figure S4a
250
130
95
72
55
36
28
17
10
kDa
250
130
95
72
55
36
28
17
10
kDa
anti-β-actin
anti-GFP
Figure S4b
250
130
95
72
55
36
28
17
10
kDa
250
130
95
72
55
36
28
17
10
kDa
anti-GFP
anti-β-actin
Figure S4c
Figure S10. Original western blotting analysis of GFP (left) or β-actin (right) presented as Figure S4a (top images), Figure S4b (middle images) and Figure S4c (bottom images).

## Slide 11
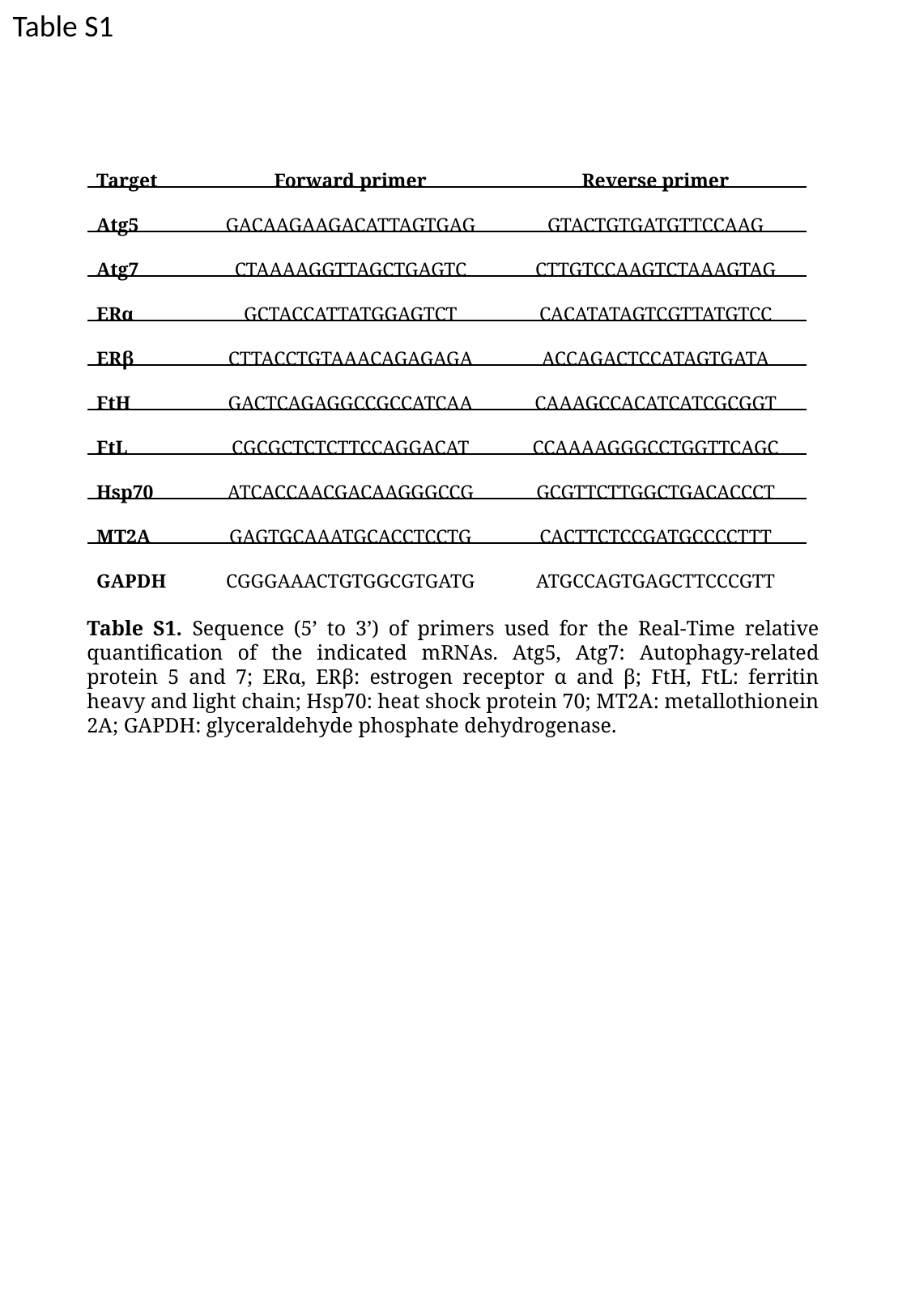

Table S1
| Target | Forward primer | Reverse primer |
| --- | --- | --- |
| Atg5 | GACAAGAAGACATTAGTGAG | GTACTGTGATGTTCCAAG |
| Atg7 | CTAAAAGGTTAGCTGAGTC | CTTGTCCAAGTCTAAAGTAG |
| ERα | GCTACCATTATGGAGTCT | CACATATAGTCGTTATGTCC |
| ERβ | CTTACCTGTAAACAGAGAGA | ACCAGACTCCATAGTGATA |
| FtH | GACTCAGAGGCCGCCATCAA | CAAAGCCACATCATCGCGGT |
| FtL | CGCGCTCTCTTCCAGGACAT | CCAAAAGGGCCTGGTTCAGC |
| Hsp70 | ATCACCAACGACAAGGGCCG | GCGTTCTTGGCTGACACCCT |
| MT2A | GAGTGCAAATGCACCTCCTG | CACTTCTCCGATGCCCCTTT |
| GAPDH | CGGGAAACTGTGGCGTGATG | ATGCCAGTGAGCTTCCCGTT |
Table S1. Sequence (5’ to 3’) of primers used for the Real-Time relative quantification of the indicated mRNAs. Atg5, Atg7: Autophagy-related protein 5 and 7; ERα, ERβ: estrogen receptor α and β; FtH, FtL: ferritin heavy and light chain; Hsp70: heat shock protein 70; MT2A: metallothionein 2A; GAPDH: glyceraldehyde phosphate dehydrogenase.
